# Editorial: Insulin and The Brain

**DOI:** 10.3389/fendo.2019.00299

**Published:** 2019-05-10

**Authors:** Mohammed Taouis, Ignacio Torres-Aleman

**Affiliations:** ^1^UMR9197 Institut des Neurosciences Paris Saclay (Neuro-PSI), University of Paris-Saclay, Orsay, France; ^2^Spanish National Research Council (CSIC), Madrid, Spain

**Keywords:** insulin, brain, cognition, insulin resistance, intranasal, neurodegenrative diseases, neuroinflammation

The role of insulin in the brain has been suggested in the late 1950's based on experiments showing that insulin was able to increase glucose uptake in spinal cord tissue, in several brain regions such as the choroid plexus the pineal gland, and in the pituitary (1). Since then, increasing evidence attributed to insulin action in the brain numerous critical roles in the control of vital physiological functions: energy homeostasis, neuronal plasticity, and growth, lipid, and glucose metabolism through the control of peripheral organs. Furthermore, the alteration of insulin action observed in insulin-resistant state or obesity is considered a risk factor for several pathologies including neurodegenerative diseases and metabolic disorders. Nowadays, many studies aim to decipher the mechanisms of insulin action in different brain regions and the related physio-pathological outputs.

The objective of the Research Topic “Insulin and the Brain” was to gather original research articles and reviews illustrating the recent advances concerning the roles of insulin in the brain. This Research Topic consists of four original articles and two mini-reviews.

Two original articles deal with the use of intranasal insulin administration and its impact on 1/metabolism and cognition, and 2/the modulation of odor sensitivity. This non-invasive method of insulin delivery has been used in humans to investigate the impact of central insulin on calorie intake. Indeed, an acute intranasal insulin administration reduces food intake in healthy males (2). Furthermore, chronic intranasal insulin administration during 8 weeks decreases body weight and fat mass in men but not in women (3). Interestingly, both acute and chronic intranasal insulin treatment ameliorate cognitive functions in healthy subjects (4, 5). Ritze et al. investigated the impact of subchronic intranasal insulin administration on metabolic and cognitive outcomes in healthy men. They aimed to differentiate between pre-meal and nocturnal chronic intranasal insulin delivery on metabolic parameters and cognition. They show that body weight and metabolic/endocrine parameters are not modified by either morning or evening insulin delivery. They report an improvement of memory performance when insulin is administered in the evening. Beside its impact on the regulation of metabolism and cognition, intranasal insulin affects olfactory processing. Actually, the augmentation of insulin levels in the cerebrospinal fluid of healthy subjects reduces olfactory sensitivity for the odorant n-butanol (6). It is noteworthy that intranasal insulin increases insulin levels in cerebrospinal fluid (7). Rodriguez-Raecke et al. investigated the influence of intranasal insulin on olfactory sensitivity using n-butanol and peanut in healthy subjects. They use a single intranasal insulin administration and show a decrease in olfactory sensitivity toward n-butanol and not peanut in females but not in males. The authors conclude that intranasal insulin administration increases cortical insulin levels, then modulating olfactory sensitivity for specific odors in a gender-dependent manner. These two articles propose the intranasal insulin delivery as a potent route to increase brain insulin levels that should be considered when cerebral insulin levels are decreased such as in insulin resistance state and reduced insulin activity in the brain in patients with type two diabetes or in obese patients. The third article deals with the impact of insulin in the hippocampus on cognitive functions in diet-induced obesity. High-fat diet (HFD) consumption increases hippocampus inflammation associated with the impairment of memory and spatial learning in mice (8–10). Gladding et al. investigated the effect of intra-hippocampal insulin infusion on spatial cognition and associated neuroinflammation in a context of diet-induced obesity. They demonstrate that HFD impairs spatial memory performance accompanied by the inflammation of the hippocampus. Importantly, they demonstrate that these deleterious effects of HFD are attenuated in mice receiving an intrahippocampal insulin infusion that also reduces the inflammation of the hippocampus. These findings may be of great interest for human subjects suffering from obesity that is now considered as a risk factor for dementia. Indeed, insulin levels in brain areas including hippocampus could be increased by intranasal insulin administration as demonstrated in the first two articles of this topic. The presented articles clearly demonstrate the importance of insulin in different brain regions implicated in cognition, modulation of odor sensitivity, and energy homeostasis by directly applying insulin in specific brain areas. Schleger et al. investigated fetal brain activity in normoglycemic mothers with or without family history of diabetes (FHD). This group has previously shown that gestational diabetes mellitus (GDM) affects human fetus brain activity measured by magnetoencephalography (fMEG) (11). Thus, fetuses of GDM mothers subjected to an oral glucose overload exhibit a longer latency of auditory event-related brain responses (fAER) as compared to fetuses of normal glucose tolerant mothers (12). Thus, the mothers' insulin resistance could be causally linked to these disorders. To determine whether fetus brain activity is modulated by genetic or/and epigenetic factors, Schleger et al. analyzed brain activity of fetuses of normal glucose tolerant mothers with or without FHD. They show that fetuses of mothers with FHD have longer fAER and that FHD is linked to fetal postprandial brain activity. These findings suggest that the disturbance of insulin responsiveness could have long term impact on offspring brain activity.

Finally, the two mini-reviews of this topic cover two aspects associated to the alteration of brain insulin signaling: Alzheimer's disease and mechanisms of diet-induced hypothalamic inflammation. Ferreira et al. discuss the mechanisms relating diabetes, obesity, and dementia, focusing on peripheral disturbances related to diabetes and obesity that may trigger AD-like pathology. Based on the purported commonality of pathogenic mechanisms in peripheral and central insulin resistance, a new hallmark of the AD brain, they comment on the potential use of anti-diabetic drugs for AD, emphasizing ongoing intranasal insulin clinical trials. In the second mini-review, Benomar and Taouis describe molecular mechanisms underlying hypothalamus inflammation and insulin resistance. They focus on a new signaling pathway implicating resistin and its receptor TLR-4. Indeed, resistin, an adipokine, has been identified as a potential link between obesity and insulin resistance. The overview presented by these authors report that hypothalamic resistin/TLR-4 signaling is a key signaling pathway implicated in the onset of hypothalamic inflammation and insulin resistance in response to inappropriate diet such as HFD.

In summary, the articles presented in the Research Topic “Insulin and the Brain” provide a valuable source of information concerning the role of insulin in different brain areas associated to various vital physiological functions.

## Author Contributions

All authors listed have made a substantial, direct and intellectual contribution to the work, and approved it for publication.

### Conflict of Interest Statement

The authors declare that the research was conducted in the absence of any commercial or financial relationships that could be construed as a potential conflict of interest.
